# Gene mining, codon optimization and analysis of binding mechanism of an aldo-keto reductase with high activity, better substrate specificity and excellent solvent tolerance

**DOI:** 10.1371/journal.pone.0260787

**Published:** 2021-12-02

**Authors:** Wei Jiang, Xiaoli Fu, Weiliang Wu

**Affiliations:** College of Chemical Engineering, Huaqiao University, Xiamen, China; GLA University, INDIA

## Abstract

The biosynthesis of chiral alcohols has important value and high attention. Aldo–keto reductases (AKRs) mediated reduction of prochiral carbonyl compounds is an interesting way of synthesizing single enantiomers of chiral alcohols due to the high enantio-, chemo- and regioselectivity of the enzymes. However, relatively little research has been done on characterization and apply of AKRs to asymmetric synthesis of chiral alcohols. In this study, the AKR from *Candida tropicalis* MYA-3404 (*C*. *tropicalis* MYA-3404), was mined and characterized. The AKR shown wider optimum temperature and pH. The AKR exhibited varying degrees of catalytic activity for different substrates, suggesting that the AKR can catalyze a variety of substrates. It is worth mentioning that the AKR could catalytic reduction of keto compounds with benzene rings, such as cetophenone and phenoxyacetone. The AKR exhibited activity on *N*,*N*-dimethyl-3-keto-3-(2-thienyl)-1-propanamine (DKTP), a key intermediate for biosynthesis of the antidepressant drug duloxetine. Besides, the AKR still has high activity whether in a reaction system containing 10%-30% V/V organic solvent. What’s more, the AKR showed the strongest stability in six common organic solvents, DMSO, acetonitrile, ethyl acetate, isopropanol, ethanol, and methanol. And, it retains more that 70% enzyme activity after 6 hours, suggesting that the AKR has strong solvent tolerance. Furthermore, the protein sequences of the AKR and its homology were compared, and a 3D model of the AKR docking with coenzyme NADPH were constructed. And the important catalytic and binding sites were identified to explore the binding mechanism of the enzyme and its coenzyme. These properties, predominant organic solvents resistance and extensive substrate spectrum, of the AKR making it has potential applications in the pharmaceutical field.

## 1 Introduction

The synthesis of chiral alcohols and their derivatives has great significance and great market demand as they are key intermediates in many important drugs, such as the anti-tumor drug Irutinib, the anti-depression drug Duloxetine, and the lipid-lowering drug Atorvastatin [1–12]. Biocatalysis has shown advantages and concerns due to its high chiral selectivity, stereoscopic and regional selectivity, environmental friendliness, mild conditions and so on. Enzymes, such as alcohol dehydrogenase, ketone reductase, etc, are the key to the synthesis of chiral compounds by biocatalysis [7–15]. Therefore, it is very important to obtain enzymes with good performance, but relatively few of them can be applied to the industrialized production of chiral compounds.

The aldo-keto reductases (AKRs) could catalyze the reduction of ketones or different acids to chiral alcohols [16–18]. The AKRs are one of the three super families that perform oxidoreduction on a variety of natural and synthetic substrates [19]. AKRs mediated stereoselective reduction of prochiral carbonyl compounds is an efficient way of preparing single enantiomers of chiral alcohols due to the high enantio-, chemo- and regioselectivity of the enzymes. But only a few of AKRs have been found, characterized, and applied to biosynthesis of chiral alcohols. A novel AKR, Tm1743, was obtained from *Thermotoga maritimeit*, and it shown broad substrate specificity towards many ketones, keto esters, and aldehydes [20]. The AKR,YOL151W, from *Saccharomyces cerevisiae* was hired to asymmetric synthesis of (*S*)-3-chloro-1-phenyl-1-propanol [21]. Besides, the AKRs from *Penicillium citrinum* IFO4631, *C*. *albicans*, and *Kluyveromyces lactis* XP1461, was also used for synthesis chiral alcohols [22–24]. Nevertheless, the living example of the synthesis of chiral alcohols by AKRs are few compared to alcohol dehydrogenase and carbonyl reductase, and, it was deserved to be studied [25, 26].

In this study, a aldo-keto reductase AKR was obtained from *C*. *tropicalis* MYA-3404, its codon was optimized and its performance was analyzed. It was showed that the AKR own high activity, organic solvent tolerance, and broad substrate spectrum, making it a promising catalyst for the biosynthesis of chiral alcohols. In addition, in order to explore the binding mechanism of the AKR with coenzyme, molecular docking was also used.

## 2 Materials and methods

### 2.1 Chemicals and strains

All chemicals are analytical or chromatographic grade, and they were purchased from Sinopharm Group or Aladdin (Germany) without further purification. All enzymes were purchased from TaKaRa Co., Ltd. (Dalian, China). The Gel Extraction Kit(200) and Plasmid Mini Kit I (200) were obtained from OMEGA Co.(USA). NADP(H) were obtained from Sigma-Aldrich Co. (Shanghai, China). *Escherichia coli* (*E*. *coli*) DH5α and *E*. *coli* BL21 (DE3) was used cloned and heterologous expression, respectively.

### 2.2 Gene discovery and homologous protein-searching analysis

The keto reductase sequence (GenBank accession number MK503097) was obtained in our laboratory. And it was used as template to search for similar target sequences in *C*. *tropicalis*. The BLAST tool, https://blast.ncbi.nlm.nih.gov/Blast.cgi, was used in this progress. As HummePankaj Soni and U. C. Banerjee reported that *Candida tropicalis* has the ability to synthesize duloxetine, but the specific protein has not been identified [27]. We hypothesized that keto reductase might play an important role in this, so the scope of retrieval was determined in the *C*. *tropicalis*.

### 2.3 Codon optimization, expression and purification of the AKR

In order to improve the protein expression level, the *akr* gene sequence was optimized according to codon preference of *E*. *coli*. The Optimized gene sequence was synthesized by Shanghai Sangon Co., Ltd., and the gene was subcloned into the vector of pET-28a, generating a recombinant expression vector pET-28a-*akr*, then it was transformed into *E*. *coli* BL21 (DE3) cells for express the recombinant protein. The *E*. *coli* BL21 (DE3) was cultured in 100 mL LB medium containing 100 μg/mL of ampicillin at 200 rpm, 37° C. The IPTG was added with a final concentration of 0.1 mM when an optical density of 600 nm (OD 600) of the culture reached 0.6–0.8, the inducing temperature was 18° C. It was cultured for another 12 hours. Then, the culture was then harvested (8000 rpm, 5 minutes), and washed three times by using PBS buffer at 4°C. The cell pellet was suspended and disrupted by using sonication (JY92-IIN ultrasonic cell crusher, Ningbo Xinzhi Biotechnology Co., LTD). After that, the disrupted cell debris was centrifuged for 30 minutes at 12,000 rpm, 4°C. Then, the tagged enzyme was purified by an AKTA Prime system equipped with a 10-mL Ni-IDA column (GE Healthcare, USA). Finally, the results and concentration of purification of the AKR were determined by 12% SDS-PAGE and Bradford Protein Assay Kit.

### 2.4 Characterization of the AKR

#### 2.4.1 Substrate spectrum of AKR

In order to investigate the substrate specificity of the AKR, the enzyme was selected to catalyzed reduction of ketones, ketoesters and aldehydes under standard conditions. One unit of AKR was defined as the content of AKR that catalyzed the oxidation of 1 μmol NADPH PM (Per Minute). The rates of reactions were assayed at indoor temperature by detecting the change in absorption peak of NADPH at 340 nm (ε = 6.22 mM^−1^ cm^−1^). The reaction mixture (220μl) contained 10 μl substrates, 170μl PBS buffer, 10μl NADPH, and 30μl enzyme AKR. The residual maximum activity was defined as 100%. The substrate were 3-methylcyclohexanone, methyl pyruvate, phenoxy ethyl ketone, ethyl levulinate, 4-octanone, acetylacetone, 5-methyl-2-hexanone, methyl isobutyl ketone, acetophenone, *N*,*N*-dimethyl-3-keto-3-(2-thienyl)-1-propanamine (DKTP).

#### 2.4.2 Effect of pH and temperature

To detect the pH eect of the AKR, the enzyme activity was measured in 50 mM buffers systems of pH 5.0–6.0 (CH_3_COOH-CH_3_COONa), 6.0–8.0 (NaH_2_PO_4_-Na_2_HPO_4_) and 8.0–9.0 (Tris-HCl buffers). To detect the optimum temperature of the AKR, the reaction system (without NADPH) was preincubated at the respective temperature for 5 minutes, then, the enzyme activity was detected in different temperature, 30–50 ℃. The substrate used for enzyme activity determination is the optimal substrate, acetylacetone, for this enzyme.

#### 2.4.3 Organic solvent tolerance of AKR

Different kinds of organic solvents (10%, 20%, 30%) V/V were introduced into the reaction system for detecting the performance of tolerate organic solvents and the effect of organic solvents on the AKR. Common organic solvents, methanol, isopropanol, ethanol, ethyl acetate, Dimethyl sulfoxideDMSO), and acetonitrile were chosen as the object of study. The residual activity of the enzyme in incubated samples was detected according to standard assay methods. Besides, the performance of tolerate organic solvents of AKR was executed by detect the residual activity of AKR in the reaction system with 10% v/v of different kinds of organic solvents for 6 hours. The original AKR’s activity was designated as 100% and the optimum substrate, acetylacetone, was selected as the substrate.

### 2.5 Molecular docking and key site resolution

The BLAST tool (NCBI, https://blast.ncbi.nlm.nih.gov/Blast.cgi) was selected to discover sequences similar to the AKR. Nine other AKRs were obtained from blast results, and in order to analyze the source of the AKR, DNAMAN was used to draw the alignment homologous tree. Homology modeling was built by using SWISS MODEL (http://swissmodel.expasy.org/) according to the sequence information of AKR [28]. In addition, the Docking service for molecular docking was employed to explore the binding mechanism of the enzyme and coenzyme.

## 3 Result and discussion

### 3.1 Gene discovery and codon optimization

**Fig 1:**
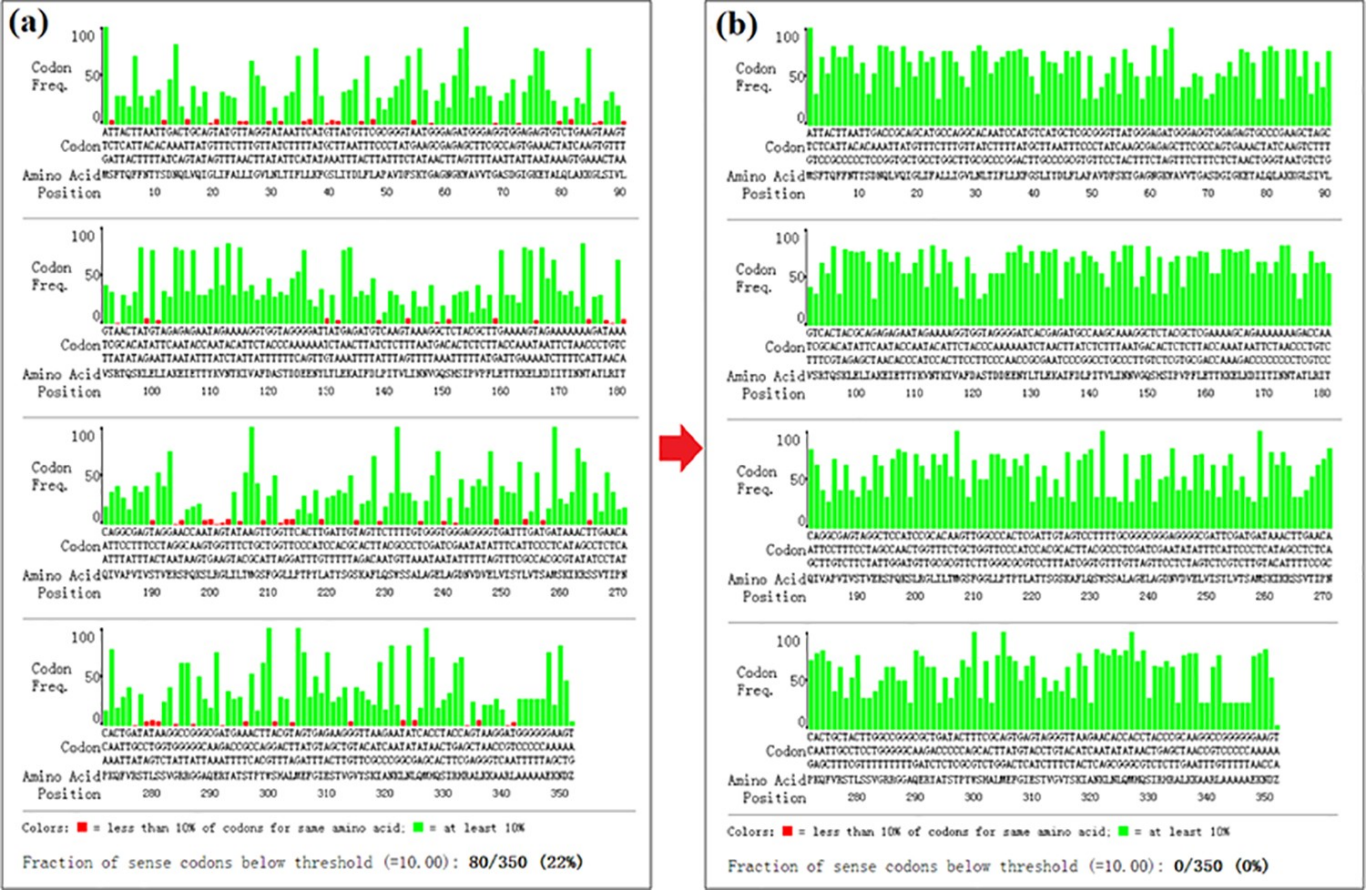
Codon optimization of the *akr* gene. (a) Before optimization; (b) After optimization.

The *C*. *tropicalis* was determined as the scope of retrieval for it shown the ability to synthesize duloxetine [27]. However, the specific protein has not been identified. And combined with other relevant studies, we hypothesized that AKR might play an important role in this progress. The AKR sequence, (GenBank accession number MK503097) which was obtained in our laboratory, were used as templates to mine the target sequences in *C*. *tropicalis*. After digging and analysis, the *akr* gene (GenBank accession number NW_003020038.1) from *C*. *tropicalis MYA-3404* was obtained. The *akr* gene sequence was optimized according to codon preference of *E*. *coli* to improve the protein expression level. The results of codon optimization are shown in Fig 1. After optimization, the frequency of rare codons was greatly reduced. And it was demonstrated that codon optimization can significantly improve the expression level of recombinant protein.

### 3.2 Substrate specificity

**Fig 2:**
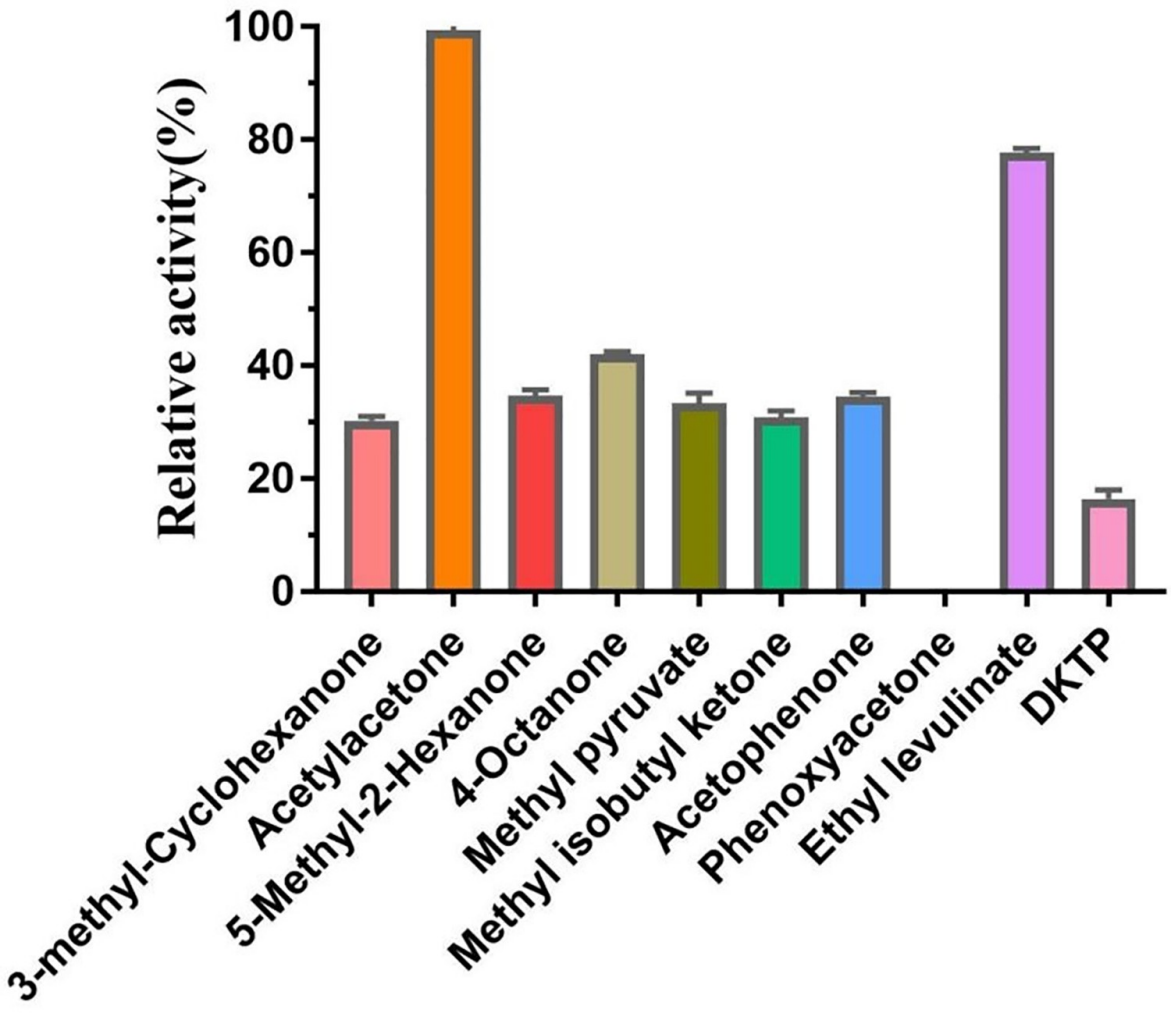
Substrate specificity of the AKR.

The substrate specificity of AKR was investigated by detecting the catalytic efficiency of AKR for various keto acids and ketones. It was shown in the Fig 2 that the AKR showed varying degrees of catalytic activity, suggesting that the AKR can catalyze a variety of substrates, but it has a good substrate specificity. The activity of acetylacetone is the highest.

It is worth mentioning that the AKR could catalytic reduction of keto compounds with benzene rings, such as acetophenone and phenoxyacetone. And, the AKR exhibited about 20% relative activity on DKTP, the key intermediate for biosynthesis of the antidepressant drug duloxetine [27, 29]. These results indicate that the AKR could be employed to synthesis of chiral alcohol compounds containing a benzene ring and duloxetine, so the enzyme has a high application potential.

### 3.3 Effect of pH and temperature

**Fig 3:**
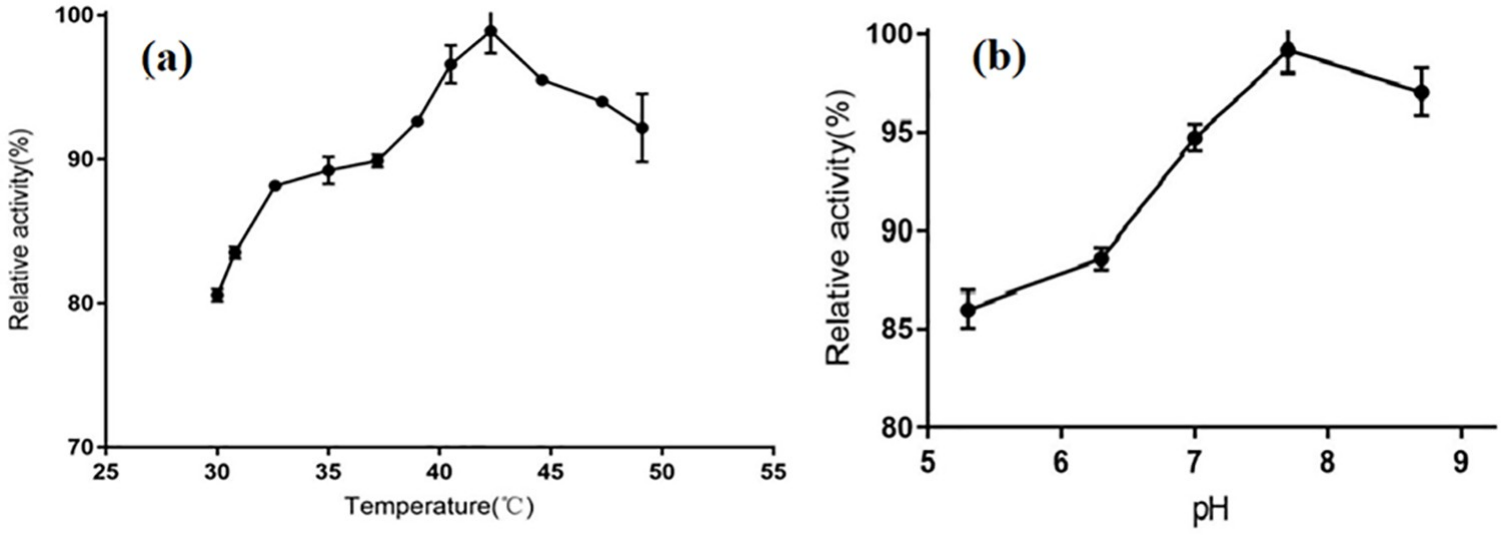
Effect of pH and temperature of the AKR. (A) Effect of temperature of the AKR; (B) Effect of pH of the AKR.

pH and temperature play an important role in the influence and regulation of enzyme activity. As shown in Fig 3A, the relative activity of the AKR was more than 80% from 30°C to 50°C. At 42.3°C, the AKR exhibited the highest activity. After the temperature exceeded to 42.3°C, the enzyme activity began to decrease. The optimum temperature for the AKR was higher than that of the aldo-keto reductase, which exhibited the highest activity at 30°C, from *Kluyveromyces lactis* XP1461 [24]. As shown in Fig 3B, the AKR has maximum activity at pH 7.7. The enzyme maintained more than 85% activity at pH 5.3–8.7, demonstrating that it has relatively high activity in a broad pH range. When the pH was 7.0–8.7, the activity of AKR was above more than 95%.

### 3.4 Organic solvent tolerance of AKR

**Fig 4:**
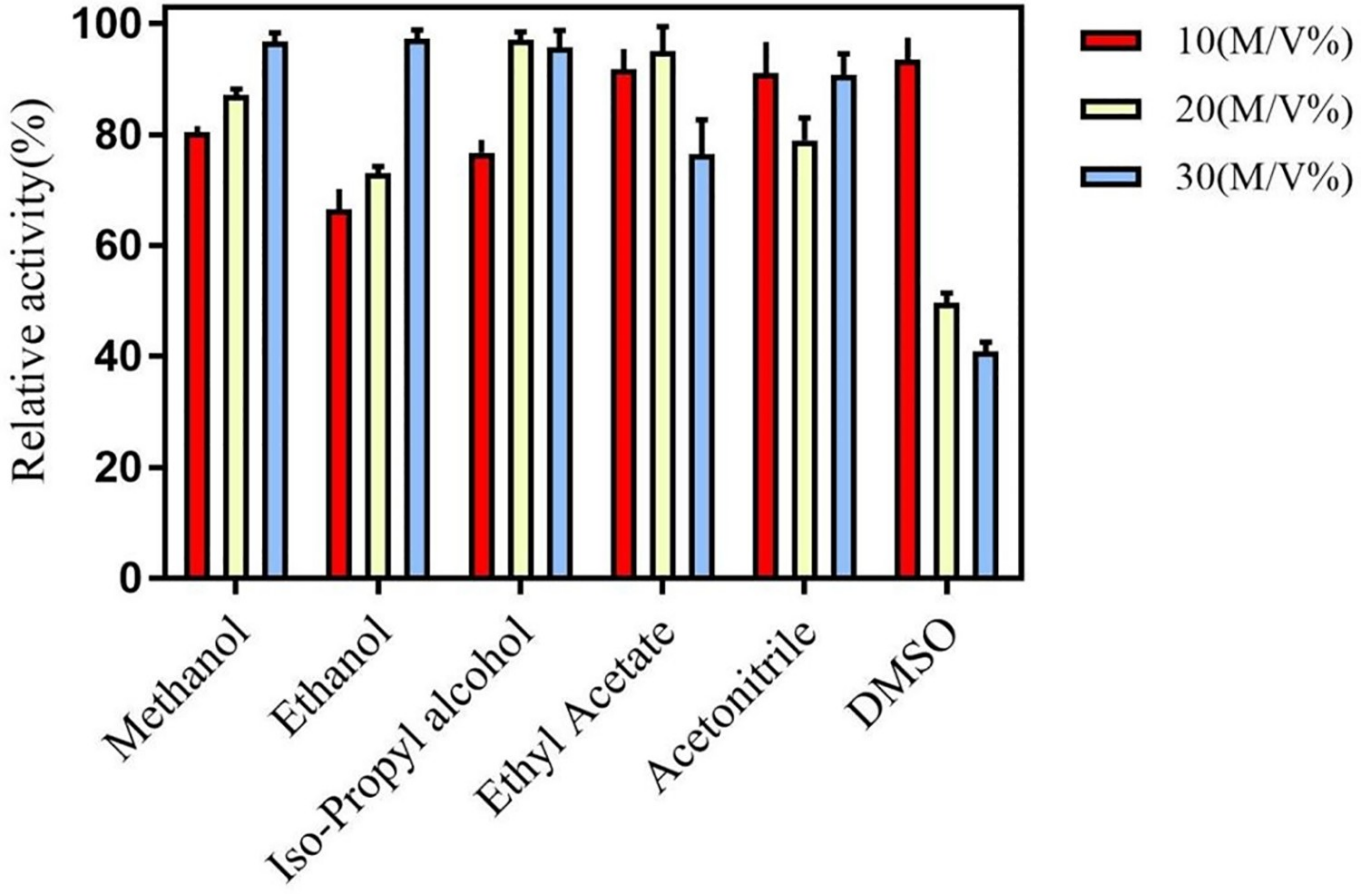
Organic solvent tolerance of the AKR. AKR is sensitive to six different organic solvents (methanol,Ethanol, 2-propanol, ethyl acetate, acetonitrile, and DMSO) with a gradient of 10%(v/v) to 30%(v/v) for each organic solvent. Three parallels were set for each experiment, and the error bar indicated the standard error of the mean value.

**Fig 5:**
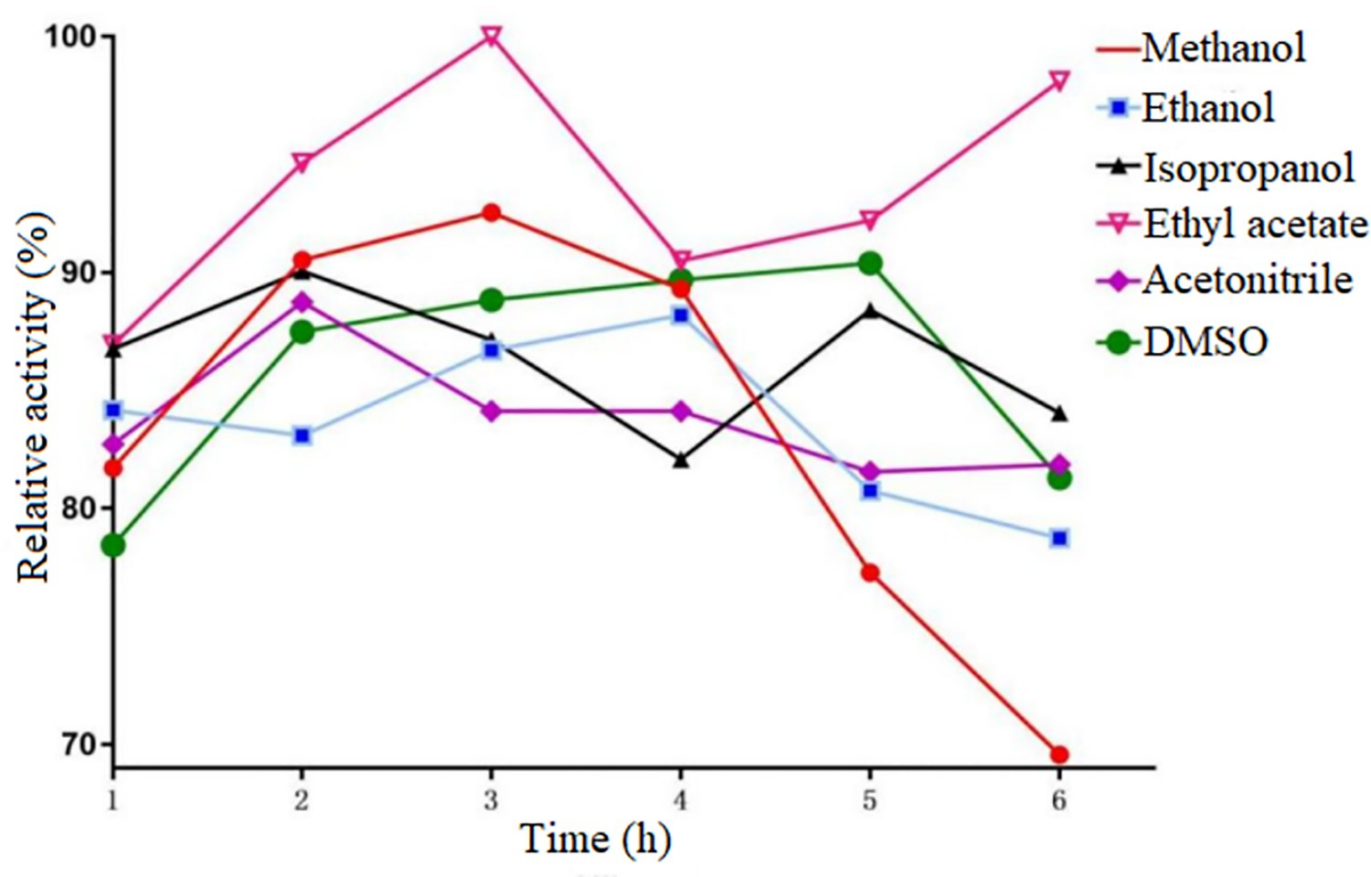
Effects of incubation time on the tolerance of AKR to organic solvents. Time 1h-6h to 6 h, six organic solvents (methanol,Ethanol, 2-propanol, ethyl acetate, acetonitrile, and DMSO), 10%(v/v) organic solvent.

Synthesis of chiral compounds are usually executed in organic solvents, so it is important that the enzyme own high organic solvent tolerance. So, six common organic solvents, DMSO, acetonitrile, ethyl acetate, isopropanol, ethanol, and methanol were selected to detect the organic solvent tolerance of the enzyme. The result was showed in Figs 4 and 5. 10%-30% (V/V) of organic solvent was added in reaction system, and the result of the activity of AKR in 10% to 30% (V/V) in the six organic solvents was showed in the Fig 4. The enzyme exhibited high activity and it all retained more than 60% activity except in the 20%-30% (V/V) of DMSO (Fig 4). The organic solvent tolerance of the enzyme was very superior compared to the aldo-keto reductase, which can only withstand 5% V/V organic solvent, from *Kluyveromyces lactis* [24]. Interestingly, the activity of the AKR increased from 10% to 30% (V/V) isopropanol, ethanol or methanol. So, it was speculated that an appropriate amount of isopropanol, ethanol or methanol can alter the spatial structure of the AKR, thereby improving enzyme activity. However, the specific reaction mechanism was not clear in this progress. So, it will be detected in the future work.

Besides, the stability of the enzyme in the organic solvents was investigated to examine the effective reaction time of the enzyme. The AKR exhibited the strongest stability in six common organic solvents, DMSO, acetonitrile, ethyl acetate, isopropanol, ethanol, and methanol (Fig 5). And, it retains more that 70% enzyme activity after 6 hours. These results indicated that the AKR own a strong organic solvent tolerance in common organic solvents, suggesting that the enzyme has high potential applications in the high value-added chemicals synthesis and biopharmaceutical fields [30–35].

### 3.5 Molecular docking and key site resolution

**Fig 6:**
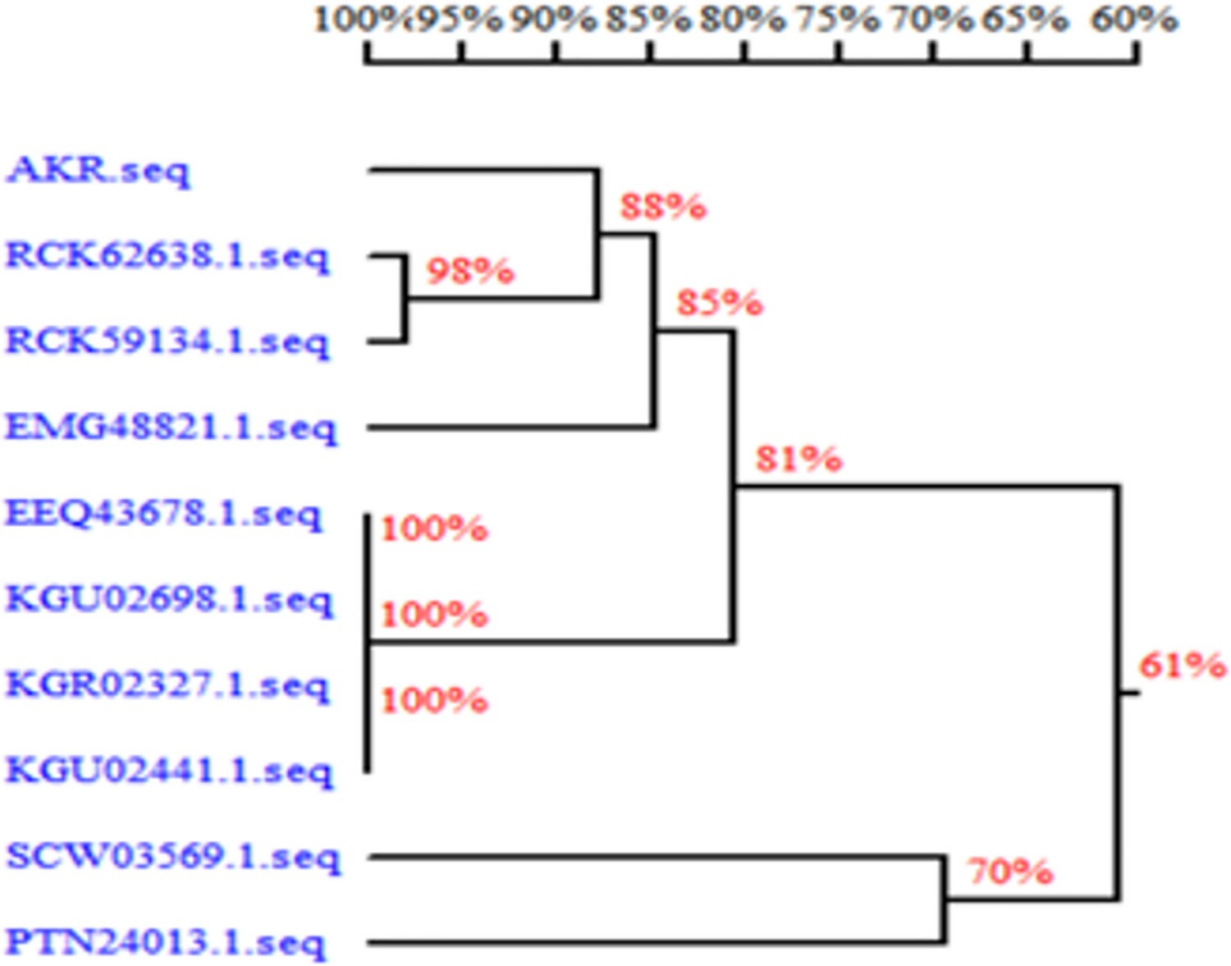
AKR sequence alignment homologous tree.

**Fig 7:**
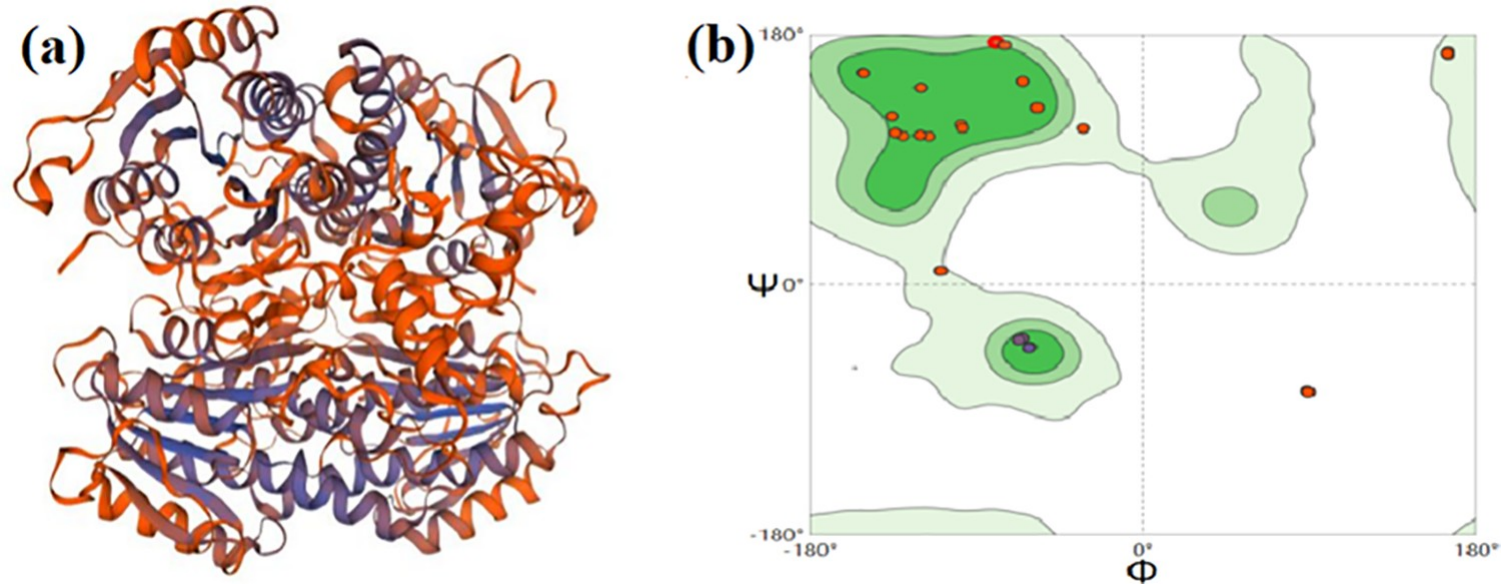
(a) The 3D model of the AKR, (b) Raman diagram for the AKR model evaluation.

The source of the AKR was analyzed and DNAMAN was used to draw the alignment homologous tree, as shown in Fig 6. The results showed that the homology between the AKR and the same kind was low. Homology modeling of the AKR was built by using SWISS-MODEL, which showed the unique three-dimensional structure (Fig 7A). The symmetric 4-subunit structure suggested that the AKR is relatively conservative. Besides, the structure of the AKR was also evaluated (Fig 7B). Key indicators of model evaluation are shown in the red dot in the Fig 7B, and it can be seen from the Fig 7B that the key evaluation factors are concentrated within a limited range. The results indicated that the three-dimensional structure model of the AKR is reliable.

**Fig 8:**
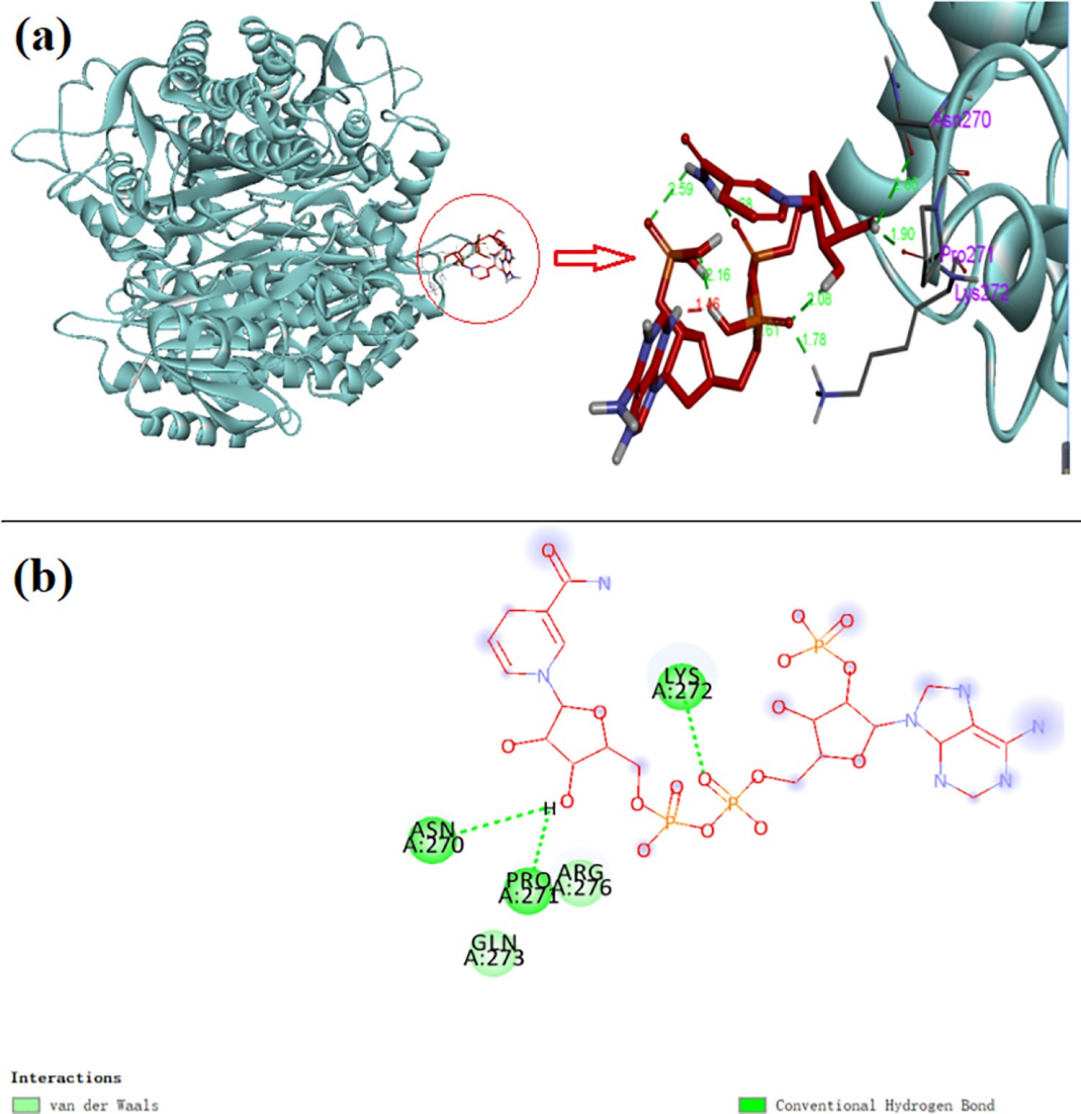
Molecular docking of the AKR with NADPH. (a) The AKR molecular docking model; (b) The AKR molecular docking local forces.

Since the AKR relies on the coenzyme NADPH to provide [H] during the reduction reaction with the substrate, so its conformation will undergo corresponding changes when the AKR combines with the coenzyme to form a holoenzyme. Through molecular docking, the amino acid residues that the AKR interacted with the coenzyme NADPH were predicted, and the molecular docking results were shown in Fig 8. As can be seen from the figure, the coenzyme binds to the tail end of the outer barrel structure of the AKR, and the amino acid residues in contact with it are Lys272, Pro271, and Asn270. Lys272 provides electrostatic stability and helps stabilize the structure of the enzyme coenzyme complex, while Pro271 and Asn270 provide REDOX reaction conditions and transfer protons [36, 37].

Unlike the common AKR 3 subunit structure, the AKR has a 4-subunit structure where NADPH binds to one of the subunits and is exposed outside the protein molecule rather than contained inside the molecule (Fig 8A). The local forces of molecular docking are shown in Fig 8B. Lys272, Pro271 and Asn270 interact with NADPH by hydrogen bonds, while Arg276 and Gln273 stabilize the enzyme coenzyme complex by van der Waals forces. Thus, the AKR may have more than one single active site.

## 4 Conclusions

A keto reductase with excellent properties was mined and characterized. The properties of the AKR were exploited and it shown wider optimum temperature and pH. Besides, the AKR has a better substrate specificity and excellent solvent tolerance. More importantly, the AKR showed activity on DKTP, an important intermediate for biosynthesis of the antidepressant duloxetine. To explore the binding mechanism of the enzyme and its coenzyme, a 3D model of the AKR docking with NADPH were constructed. Then, the important catalytic and binding sites were identified. These properties of the AKR demonstrated that it has potential applications in the field of chiral drug synthesis.

## Supporting information

S1 Data (XLSX)
